# Supplementary figures and images for: Benchmarking a targeted 16S ribosomal RNA gene enrichment approach to reconstruct ancient microbial communities
Source: PeerJ. 2024 Mar 1;12:e16770. doi: 10.7717/peerj.16770 (PMC10911074; doi:10.7717/peerj.16770)

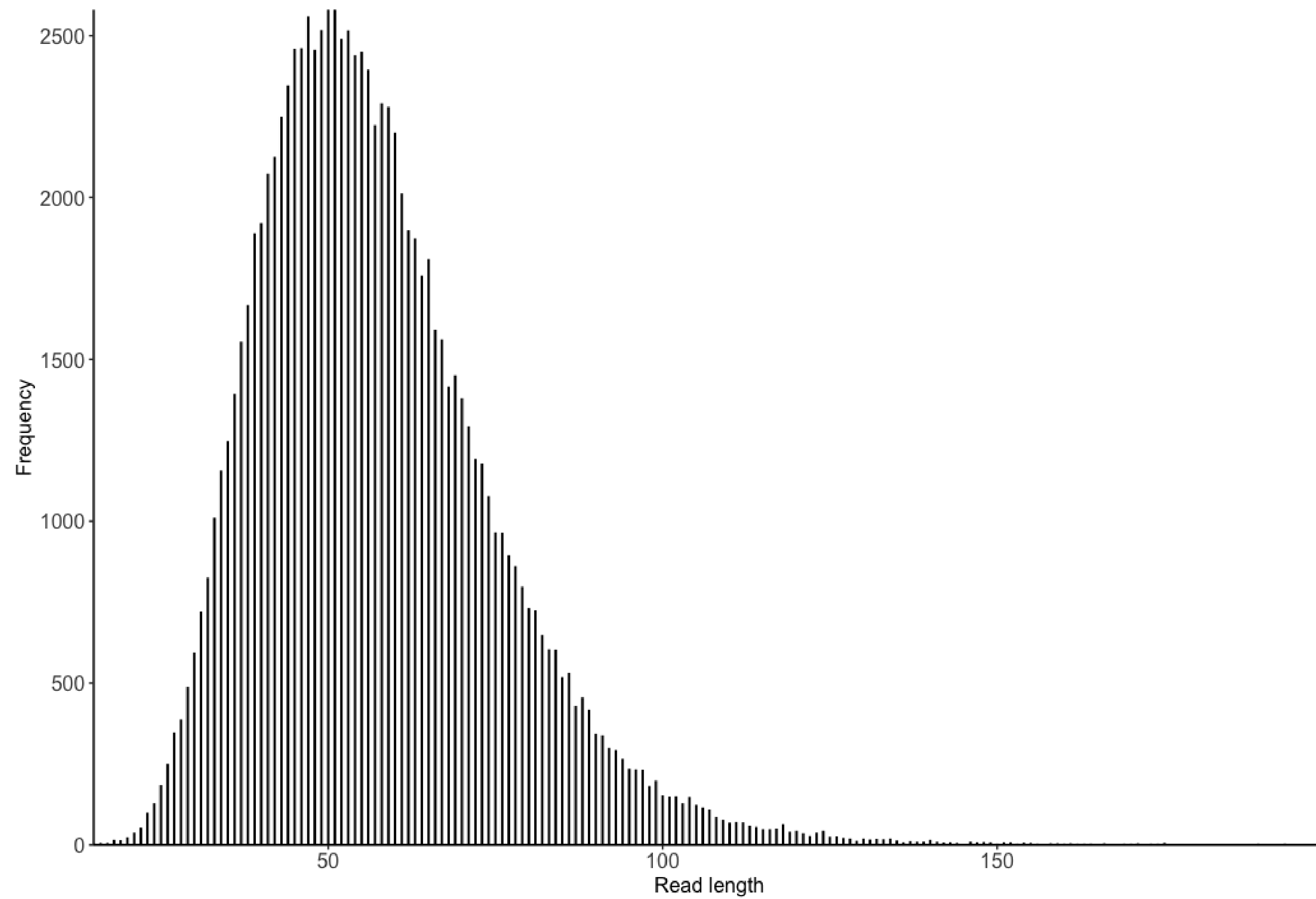

Supplement: Supplemental Information 1 [file peerj-12-16770-s001.pdf]

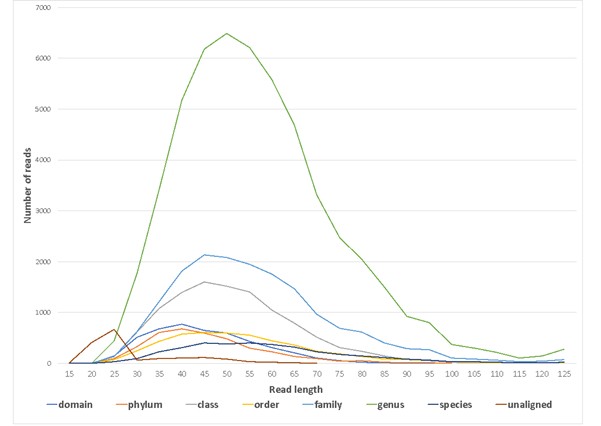

Supplement: Supplemental Information 2 [file peerj-12-16770-s002.png]

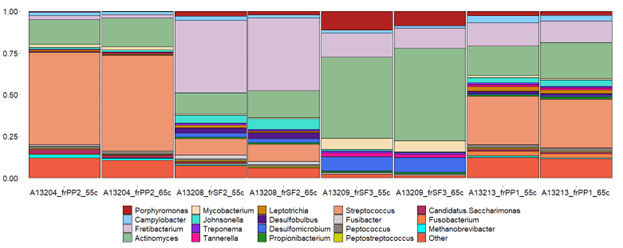

Supplement: Supplemental Information 3 [file peerj-12-16770-s003.png]

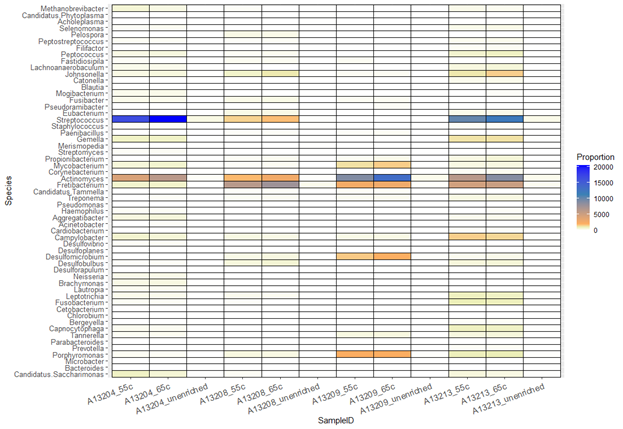

Supplement: Supplemental Information 4 [file peerj-12-16770-s004.png]

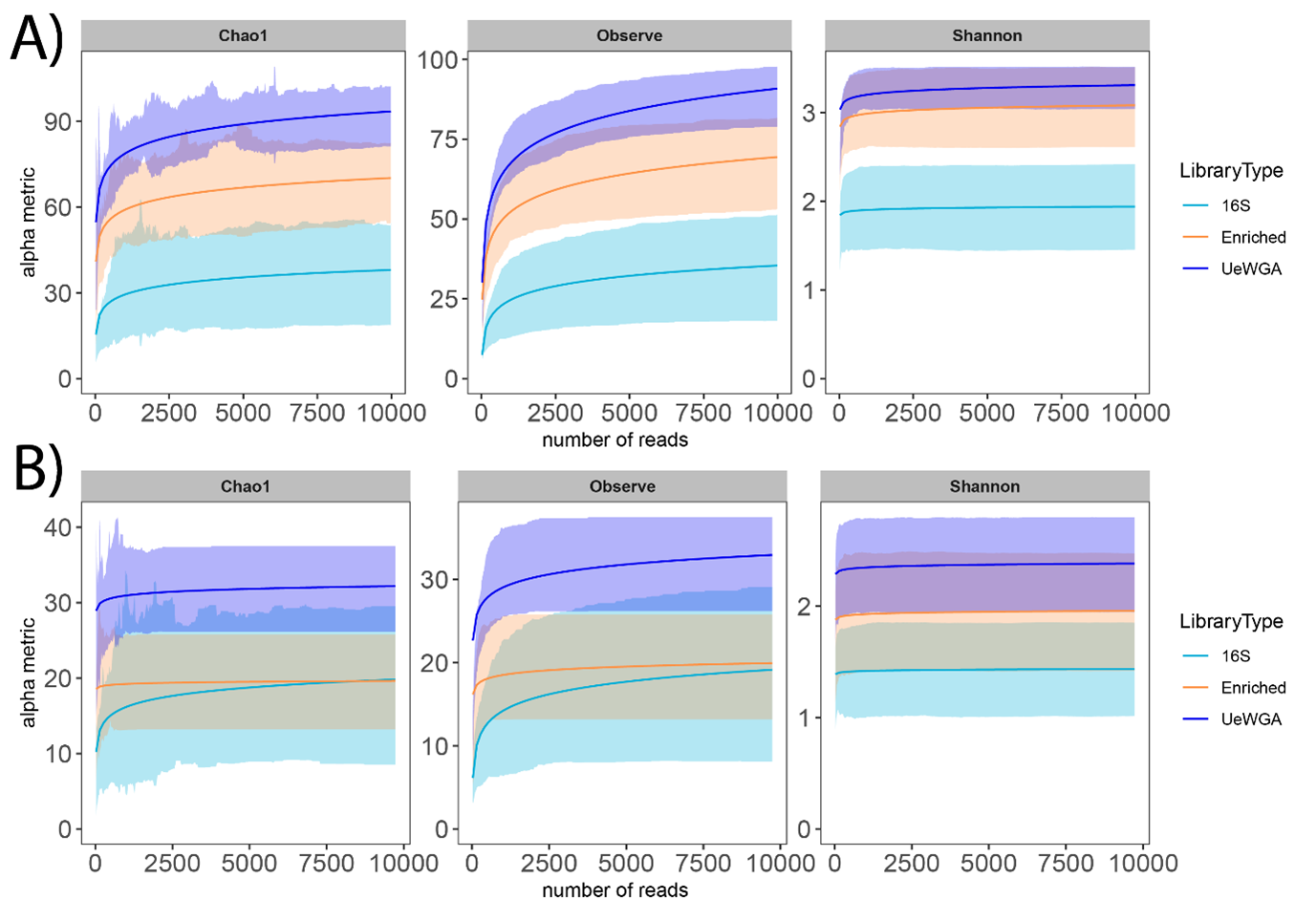

Supplement: Supplemental Information 5 — A) Shows Chao1, Observe, and Shannon results at the feature level, whereas B) shows the results at the genus level. Samples for this analysis were rarefied using the rarefy_even_depth function with the following parameters: rngseed = 1, sample.size = 0.9* min (sample_sums(), replace = F). [file peerj-12-16770-s005.png]
